# Student academic performance factors affecting matching into first-choice residency and competitive specialties

**DOI:** 10.1186/s12909-019-1669-9

**Published:** 2019-07-01

**Authors:** Katherine Mitsouras, Fanglong Dong, Marian N. Safaoui, Scott C. Helf

**Affiliations:** 1 Department of Anatomy, College of Osteopathic Medicine of the Pacific, Western University of Health Sciences, Pomona, CA 91766-1854 USA; 2 Graduate College of Biomedical Sciences, Western University of Health Sciences, Pomona, CA 91766-1854 USA; 3 Academic Informatics, College of Osteopathic Medicine of the Pacific, Western University of Health Sciences, Pomona, CA 91766-1854 USA

**Keywords:** Licensing exams, First-choice residency, Specialty, Matching

## Abstract

**Background:**

Although specific specialties and residency programs have investigated student performance factors affecting matching, there is a paucity of information from medical schools. Furthermore, factors contributing to matching into first-choice residency have not been examined. This study aimed to identify academic performance factors affecting matching into first-choice residency and highly competitive specialties.

**Methods:**

The authors conducted a study of 1726 graduates from their institution from 2010 to 2017 and assessed pre−/post-admission academic variables associated with matching into first choice and highly competitive specialties.

**Results:**

53.9% of graduates matched into their first choice. This was associated with passing COMLEX Level 2 CE (*p* = 0.01), PE (*p* = 0.02) on first attempt, and higher COMLEX Level 2 CE and USMLE 2 CK scores (*p* < 0.001 and 0.002; 14.1 and 3.9-point difference in mean scores respectively). Pre-clinical GPA (*p* = 0.002) and highest MCAT score (*p* = 0.02) were associated, however differences in means were < 1 point for both. Factors associated with matching into first choice included: MCAT (OR 0.95, 95% CI = (0.92, 0.98)), Level 2 CE score (OR = 1.01, 95% CI = (1.01, 1.02)) and passing Level 2 PE (OR = 3.68, 95% CI = (1.2, 11.28)). 12% of graduates matched into high- and 63% into low-competitiveness specialties. Matching into highly competitive specialties was associated with passing COMLEX Level 1 (*p* < 0.001), Level 2 CE (*p* < 0.001), USMLE Step 1 (*p* < 0.001) and Step 2 CK (*p* = 0.03) on first attempt. Mean scores of students matching into high- versus low-competitiveness specialties differed as follows: COMLEX Level 1 62.7 points, Level 2 CE 50.5 points, USMLE Step 1 13.6 points, Step 2 CK 7 points (all *p* < 0.001), as did pre-clinical GPA (2.4 points, *p* < 0.001). Level 1 score was the strongest predictor for matching into highly competitive specialties (OR = 1.04, 95% CI = (1.02, 1.05)).

**Conclusions:**

Licensing exam performance is important for matching into first-choice residency and into highly competitive specialties. Differences in exam scores were more pronounced for matching into highly competitive specialties than into first choice, with a larger difference in mean scores between students matching into specialties of high versus low competitiveness, than between students matching into their first- versus non first-choice residency. These results may help faculty prepare students and inform curriculum design to improve matching.

## Background

For medical students, matching into their desired specialty and first-choice residency program is the culmination of a significant amount of planning, effort, time, and money. Numerous factors contribute to a successful match into a student’s preferred specialty and residency program. These encompass quantitative (such as licensing exam scores and grades in clerkships), and qualitative metrics (such as interviews and personal statements), that residency program directors utilize to evaluate and rank applicants. Additional factors that affect residency selection and matching are subjective criteria that students use to choose a specific specialty and first-choice program, such as work hours and income for the specialty, as well as location and reputation of the program [1].

In the United States, the path to becoming a fully licensed physician starts with completing a four-year undergraduate program leading to a bachelor’s degree and taking the Medical College Admission Test (MCAT), which is a requirement for admission to medical school. Students can apply to and attend either an allopathic medical school that leads to the Medical Doctor (MD) degree, or an osteopathic medical school that awards a Doctor of Osteopathic medicine (DO) degree. While training in both MD and DO programs is 4 years and very similar in content (with the exception of Osteopathic Manipulative Treatment that DO students learn), national licensing examination requirements differ. Students in MD-granting institutions are required take the United States Medical Licensing Exam (USMLE) series whereas DO students must take the Comprehensive Medical Licensing Examination (COMLEX) series. In order to graduate and enter graduate medical education, MD students are required to pass the USMLE Step 1, Step 2 Clinical Knowledge (CK) and Step 2 Clinical Skills (CS) exams. Similarly, DO students are required to pass the COMLEX Level 1, Level 2 Cognitive Evaluation (CE) and Level 2 Physical Examination (PE) exams in order to graduate and enter graduate medical education. During their final year of study, both osteopathic and allopathic medical students apply to residency programs where they can specialize. These programs can last anywhere from three to 7 years. Allopathic students apply to programs accredited by the Accreditation Council of Graduate Medical Education (ACGME), whereas osteopathic students can apply to ACGME-accredited programs, as well as to programs accredited by the American Osteopathic Association (AOA). Although not required, osteopathic medical students can take the USMLE Step 1, and Step 2 CK exams, particularly if they apply to ACGME-accredited residency programs, since some program directors do not use COMLEX scores to select candidates to interview [2]. During the first year of graduate medical education, residents must take and pass the last part of their respective licensing exams (USMLE Step 3 for MDs and COMLEX Level 3 for DOs) to obtain a license to practice medicine in any of the 50 states in the US.

The National Resident Matching Program (NRMP) administered by the American Association of Medical Colleges (AAMC) publishes a detailed yearly report of match data for applicants to programs accredited by ACGME [2]. Similarly, the American Association of Colleges of Osteopathic Medicine (AACOM) publishes a report of match data for residencies accredited by AOA [3]. Collectively, these data reveal year-to-year changes in the match, which in turn reflect the desirability and competitiveness of specific specialties among applicants.

In addition to the AAMC and AACOM reports, there are several publications focusing on attributes of applicants that successfully match into specific specialties [4–7]. These studies examine academic performance characteristics, such as research experiences and licensing exam scores, with the goal of identifying predictors of a successful match. A secondary goal of some of these publications is to assess the competitiveness of the specialty, which is at least in part defined by the quality and number of applicants. Collectively, these studies have demonstrated the importance of USMLE Step 1 and Step 2 CK scores for matching into highly competitive specialties. [4, 7–9]. This information is extremely useful for faculty to advise students how to maximize their chance of a successful match into their first-choice residency and desired specialty.

In contrast to the abundance of such publications, there are virtually no published studies from individual medical schools examining the characteristics of graduating seniors that successfully match into different specialties or into their first-choice residency. Although some Schools of Medicine (SOMs) and Colleges of Osteopathic Medicine (COMs) share information on the percentage of students that match into their first choice, and AAMC releases the percentage of MD seniors who match into their first choice yearly, there is a lack of published studies from individual SOMs or COMs [10, 11]. Such information can help faculty better advise and prepare students, inform curriculum design to achieve successful matching and, importantly, shed light on student risk factors for not matching. Finally, understanding the factors that lead to a successful match into first-choice residency and desired specialty is particularly timely in light of the implementation of the single accreditation system for Graduate Medical Education (GME), which will be complete by 2020 [12]. Many believe that unified accreditation will increase competition for osteopathic medical students and, consequently, might negatively affect their ability to match into more competitive, non-primary care specialties [13, 14]. Therefore, a thorough analysis of student academic performance factors that affect matching into first-choice residency and into different specialties will be particularly useful for DO-granting institutions, whose students are most likely to be affected by unified accreditation.

We aimed to identify student academic performance variables associated with matching into first-choice residency and into highly competitive specialties from the perspective of one osteopathic medical school.

## Methods

### Data collection

This was a retrospective cohort study of 1726 students that completed the DO degree requirements at Western University of Health Sciences and matched into a residency program from August 2010 through May 2017. The study protocol was deemed exempt by the Western University of Health Sciences Institutional Review Board.

Gender, self-reported ethnicity and pre- and post-admissions student academic performance data were collected for all study participants. Pre-admissions data consisted of highest Medical College Admission Test (MCAT) score, undergraduate science Grade Point Average (GPA), undergraduate overall GPA and Barron’s selectivity rating of the institution where undergraduate degree was obtained. The version of the MCAT used in this study is the one prior to 2015, since this version was accepted by the admissions office for students matriculating during the eight-year period of the study. The 2017 Barron’s undergraduate institutional selectivity ratings were classified as follows [15]: 0 not ranked, 1 non-competitive, 2 less competitive, 3 competitive, 4 competitive +, 5 very competitive, 6 very competitive +, 7 highly competitive, 8 highly competitive +, 9 most competitive. Post-admissions academic performance data consisted of pre-clinical GPA, Comprehensive Osteopathic Medical Licensing Exam (COMLEX) Level 1 and Level 2 Clinical Examination (CE) scores, as well as pass/fail on the first attempt for COMLEX Level 1, 2 CE and Level 2 Physical Examination (PE; a pass/fail exam). USMLE Step 1 and Step 2 CK scores and pass/fail on the first attempt were included in the analysis for students that opted to take these exams, which are not required for graduation at our institution.

All data were collected into our commercially available system, ProgressIQ™, retrieved, anonymized and analyzed in aggregate. Self-reported data on residency match, specialty as well as on whether or not each graduate matched into their first-choice residency was collected each year via a mandatory graduation survey of all graduates (100% response rate). Graduates had the option to decline to answer or leave blank the question on whether they matched into their first-choice residency. Those that did so were excluded from all further analyses. Additionally, respondents who did not report residency and specialty information were excluded from all analyses on specialty competitiveness. Residency match as well as specialty information was verified by the Office of the Registrar at our institution. All student identifying information was removed prior to data analysis to protect student privacy. Specialties were annotated as high-, medium- or low-competitiveness using AAMC Careers in Medicine® [16], which classify them into three competitiveness levels: 1) high: anesthesiology, child neurology, dermatology, neurological surgery, ophthalmology, orthopedic surgery, otolaryngology, otolaryngology and facial plastic surgery, physical medicine and rehabilitation, radiation oncology, 2) medium: emergency medicine, emergency medicine/internal medicine, emergency medicine/pediatrics, internal medicine/pediatrics, neurology, obstetrics and gynecology, psychiatry, diagnostic radiology, general surgery, urology, and 3) low: family medicine, family medicine/community medicine, family medicine/emergency medicine, family medicine/internal medicine, family medicine/preventive medicine, family medicine/psychiatry, integrated family medicine/neuromusculoskeletal medicine, internal medicine, internal medicine/research, anatomical and clinical pathology, pediatrics, pediatrics/medical genetics, primary care medicine, traditional rotating internship.

### Statistical analysis

All statistical analyses were performed using the SAS (Statistical Analysis System) software for Windows version 9.3 (Cary, North Carolina). Descriptive statistics were presented as means and standard deviations for continuous variables, along with frequencies and proportions for categorical variables. Independent t-tests were conducted to identify if continuous variables are different between students that matched into their first-choice versus not their first-choice residency program. An analysis of variance was conducted to assess if continuous variables were different among students that matched into specialties of low-, medium-, or high-competitiveness. Chi-square analyses were performed to identify the associations between categorical variables and residency match. Binary logistic regression analyses were conducted to identify significant factors associated with matching into first-choice residency. Ordinal logistic regression analyses were performed to identify significant factors associated with matching into residencies of medium- or high-competitiveness. The choice of possible factors for inclusion was made through bivariate analyses. The determination of possible significant factors was set at the 0.1 significance level. All other *p*-values were set at 0.05 level. All statistical analyses were two-sided.

## Results

We included a total of 1726 students in this study. A summary of the demographic, and of pre- and post-admission academic performance data is presented in Table 1. There was an equal distribution of males (51.2%;) and females (48.8%), and a small number of students self-identifying as Hispanic or Latino (3.7%). Additionally, 68% of incoming students obtained their undergraduate degree from an institution with a Barron’s institutional selectivity rating ≥ 5. The vast majority of students passed the COMLEX Level 1 (95.2%; mean score 514.2), as well as the COMLEX Level 2 CE (94%; mean score 522.5) and Level 2 PE (98.9%) national licensing exams on the first attempt. In contrast, since passing the USMLE Step 1 and Step 2 CK national licensing exams on the first attempt is not a graduation requirement, 77.7% of students took Step 1, with 88.5% of first-time exam takers passing (mean score 213.4), and even fewer (53.7%) took Step 2 CK, with 92.7% passing on the first attempt (mean score 228.1). Additionally, 53.9% of graduates self-reported matching into their first-choice residency and 63% matched into specialties that are considered low competitiveness.

**Table 1:** Demographic and academic characteristics of the DO 2010–2017 students included in the study

Characteristic		N (%)^a^
Demographics		
Gender (*n* = 1726)	Male	884 (51.2%)
	Female	842 (48.8%)
Ethnicity (*n* = 1640)	Hispanic or Latino	61 (3.7%)
	Not Hispanic or Latino	1579 (96.3%)
Pre-admission performance variables		
Barron’s selectivity rating of undergraduate institution (*n* = 1701)	0 not ranked	60 (3.5%)
	1 non-competitive	2 (0.1%)
	2 less competitive	36 (2.1%)
	3 competitive	287 (16.9%)
	4 competitive +	161 (9.5%)
	5 very competitive	340 (20.0%)
	6 very competitive +	63 (3.7%)
	7 highly competitive	180 (10.6%)
	8 highly competitive +	142 (8.4%)
	9 most competitive	430 (25.3%)
Highest MCAT score	27.9 (2.9)	
Science GPA	3.5 (0.3)	
Overall GPA	3.5 (0.2)	
Post-admissions performance variables		
Pre-clinical GPA	86 (3.8)	
COMLEX Level 1 (*n* = 1723)	514.2 (76)	
	Pass	1641 (95.2%)
	Fail	82 (4.8%)
COMLEX Level 2 CE (*n* = 1723)	522.5 (88.1)	
	Pass	1620 (94.0%)
	Fail	103 (6.0%)
COMLEX Level 2 PE (*n* = 1723)	N/A	
	Pass	1704 (98.9%)
	Fail	19 (1.1%)
USMLE Step 1 (*n* = 1341)	213.4 (19.6)	
	Pass	1187 (88.5%)
	Fail	154 (11.5%)
USMLE Step 2 CK (*n* = 926)	228.1 (18.6)	
	Pass	858 (92.7%)
	Fail	68 (7.3%)
Residency match		
First vs not first choice (*n* = 1726)	First	931 (53.9%)
	Not first	795 (46.1%)
Specialty competitiveness (*n* = 1664)	High	199 (12.0%)
	Medium	416 (25.0%)
	Low	1049 (63.0%)

^a^All values in Table 1 are presented as N (%) or as Mean (Standard Deviation)

Our first objective was to examine pre- or post-admissions student academic performance variables associated with matching into first-choice residency. Of the 13 variables we considered, six were significantly associated (shown in bold in Table 2). The only pre-admissions variable associated with matching into first-choice residency was the highest MCAT score, with a 0.4-point difference (on a 45-point score scale) in mean scores between the two groups (*p* = 0.02). Surprisingly, the mean highest MCAT score of those that matched into their first choice was 0.4 points lower than that of those that did not. Additionally, the Barron’s undergraduate institutional selectivity ratings were roughly equally distributed among those matching into their first choice versus those that did not.

**Table 2:** Pre- and post-admission academic variables affecting matching into first-choice residency for DO 2010–2017

Factor	First-choice	Not first-choice	*p*-value^a^
Pre-admission performance variables			
Barron’s selectivity rating of undergraduate institution (*n* = 1701)			N/A
0 not ranked	37 (61.7%)	23 (38.3%)	
1 non-competitive	1 (50%)	1 (50%)	
2 less competitive	19 (52.8%)	17 (47.2%)	
3 competitive	172 (59.9%)	115 (40.1%)	
4 competitive +	73 (45.3%)	88 (54.7%)	
5 very competitive	186 (54.7%)	154 (45.3%)	
6 very competitive +	29 (46%)	34 (54%)	
7 highly competitive	93 (51.7%)	87 (48.3%)	
8 highly competitive +	70 (49.3%)	72 (50.7%)	
9 most competitive	237 (55.1%)	193 (44.9%)	
Mean Highest MCAT Score	**27.7 (2.9)**	**28.1 (2.8)**	**0.02**
Science GPA	3.5 (0.3)	3.5 (0.3)	0.99
Overall GPA	3.5 (0.2)	3.5 (0.2)	0.53
Post-admissions performance variables			
Pre-clinical GPA	**86.3 (3.7)**	**85.7 (3.9)**	**0.002**
COMLEX Level 1 (*n* = 1723)			
Score	517.2 (76.2)	510.7 (75.7)	0.08
Pass/Fail			0.87
Pass	885 (53.9%)	756 (46.1%)	
Fail	45 (54.9%)	37 (45.1%)	
COMLEX Level 2 CE (*n* = 1723)			
Score	**529 (89.3)**	**514.9 (86.1)**	**< 0.001**
Pass/Fail			**0.01**
Pass	887 (54.8%)	733 (45.3%)	
Fail	43 (41.8%)	60 (58.3%)	
COMLEX Level 2 PE Pass/Fail (*n* = 1723)			**0.02**
Pass	925 (54.3%)	779 (45.7%)	
Fail	5 (26.3%)	14 (73.7%)	
USMLE Step 1 (*n* = 1341)			
Score	213.3 (20)	213.6 (19)	0.85
Pass/Fail			0.08
Pass	619 (52.2%)	568 (47.9%)	
Fail	92 (59.7%)	62 (40.3%)	
USMLE Step 2 CK (*n* = 926)			
Score	**230.1 (18)**	**226.2 (19)**	**0.002**
Pass/Fail			0.09
Pass	432 (50.4%)	426 (49.7%)	
Fail	27 (39.7%)	41 (60.3%)	

^a^ All values in Table 2 are presented as N (%) or as Mean (Standard Deviation). Statistically significant associations are shown in bold

Among the post-admissions performance variables, the mean pre-clinical GPA of students matching into their first choice was slightly higher than that of students that did not (0.6-point difference between the two groups on 100-point GPA scale, *p* = 0.002). Passing COMLEX Level 2 CE as well as Level 2 PE on the first attempt were associated with matching into first-choice residency (*p* = 0.01 and *p* = 0.02 respectively). Furthermore, the COMLEX Level 2 CE score was a significant factor for matching into first choice. There was a 14.1-point difference (on a 990-point score scale) in mean Level 2 CE scores between students that matched into their first choice and those that did not (*p* < 0.001). Finally, we identified a small, but statistically significant difference in mean USMLE Step 2 CK scores between students that matched into their first choice and those that did not (3.9 points on a 300-point score scale; *p* = 0.002).

To examine the effect of multiple variables, a multivariate analysis was conducted using a subset of the statistically significant variables identified in Table 2. The results of this analysis are shown in Table 3. Even though USMLE Step 2 CK score was associated with matching into first-choice residency, taking the USMLE Step 2 CK exam is optional rather than a graduation requirement for our students, and including it would greatly reduce our sample size. Thus, we omitted USMLE Step 2 CK from this analysis. Similar to the univariate analysis, we observed an effect of mean highest MCAT score, with the unadjusted odds of matching into first-choice residency decreasing by 19% for every 5-point increase in score. This effect persisted when adjusting for the presence of the additional factors, with the odds of matching into first choice decreasing by 5% per 5-point increment in highest mean MCAT score. Among the post-admissions performance factors considered, we identified a small effect of COMLEX Level 2 CE scores. For every 5-point increment in COMLEX Level 2 CE score, the odds of matching into first choice increased by 1% both when considering it alone, and when adjusting for the presence of the additional variables. The largest effect was observed for COMLEX Level 2 PE, where passing increased the odds of a match into first choice by 232% when considered alone, and by 268% when accounting for the presence of the remaining factors included in the analysis. Finally, we determined that pre-clinical GPA had a small effect, increasing the odds of matching into first choice by 22% for every 5-point increment. However, this effect did not persist when including all five performance factors in the analysis.

**Table 3:** Multivariate analysis of select variables affecting matching into first-choice residency for DO 2010–2017

Factor	Unadjusted Odds Ratio	Adjusted Odds Ratio^a^
Pre-admission performance variables		
Mean Highest MCAT Score (increment = 5)	0.81 (0.69, 0.96)	0.95 (0.92, 0.98)
Post-admissions performance variables		
Pre-clinical GPA (increment = 5)	1.22 (1.08, 1.39)	Not significant
COMLEX Level 2 Score (increment = 5)	1.01 (1, 1.02)	1.01 (1.01, 1.02)
COMLEX Level 2 PE (Pass versus Fail)	3.32 (1.19, 9.26)	3.68 (1.2, 11.28)

^a^All values in Table 3 are presented as odds ratio (95% confidence interval). Only statistically significant results are shown (*p* < 0.05)

We also investigated factors that affect matching into specialties of different competitiveness. The results are summarized in Table 4, with statistically significant associations shown in bold. Passing COMLEX Level 1 and 2 CE on the first attempt increased the chance of matching into a highly competitive specialty (*p* < 0.001 for both). Specifically, 12.6% of students that passed Level 1 on the first attempt matched into a highly competitive specialty, as compared to 0% of those who failed it. Similarly, 12.4% of those passing Level 2 CE on the first attempt matched into a highly competitive specialty, while only 4.4% of those who failed it did so. The difference in mean scores between students that matched into specialties of high- versus low-competitiveness was 62.7 points for COMLEX Level 1 and 50.5 points for COMLEX Level 2 CE (on a 990-point score scale for both; *p* < 0.001 for both).

**Table 4:** Pre- and post-admission academic factors affecting matching into specialties of different competitiveness for DO 2010–2017

Factor	Competitiveness			*p*-value^a^
	High	Medium	Low	
Pre-admission performance variables				
Barron’s selectivity rating of undergraduate institution (*n* = 1641)				N/A
0 not ranked	5 (8.6%)	14 (24.1%)	39 (67.2%)	
1 non-competitive	0 (0%)	0 (0%)	2 (100%)	
2 less competitive	4 (11.8%)	7 (20.6%)	23 (67.7%)	
3 competitive	28 (10.3%)	75 (27.7%)	168 (62%)	
4 competitive +	17 (10.8%)	40 (25.3%)	101 (63.9%)	
5 very competitive	37 (11.4%)	86 (26.5%)	201 (62%)	
6 very competitive +	9 (14.8%)	15 (24.6%)	37 (60.7%)	
7 highly competitive	29 (16.4%)	51 (28.8%)	97 (54.8%)	
8 highly competitive +	23 (16.7%)	23 (16.7%)	92 (66.7%)	
9 most competitive	43 (10.3%)	102 (24.4%)	273 (65.3%)	
Mean Highest MCAT Score	28.3 (3.1)	27.9 (2.8)	27.8 (2.8)	0.08
Mean Science GPA	3.5 (0.2)	3.5 (0.3)	3.5 (0.3)	0.01
Mean Overall GPA	3.5 (0.2)	3.5 (0.2)	3.5 (0.2)	0.41
Post-admissions performance variable				
Mean pre-clinical GPA	**88** **(3.5)**	**86.3 (3.6)**	**85.6 (3.8)**	**<.001**
COMLEX Level 1 (*n* = 1661)				
Mean Score	**562.7 (73.9)**	**528.3 (74.1)**	**500 (72)**	**<.001**
Pass/Fail				**<.001**
Pass	199 (12.6%)	405 (25.6%)	979 (61.8%)	
Fail	0 (0%)	11 (14.1%)	67 (85.9%)	
COMLEX Level 2 CE (*n* = 1661)				
Mean Score	**560.3 (92.8)**	**542.7 (89.4)**	**509.8 (81.9)**	**<.001**
Pass/Fail				**<.001**
Pass	195 (12.4%)	406 (25.8%)	970 (61.7%)	
Fail	4 (4.4%)	10 (11.1%)	76 (84.4%)	
COMLEX Level 2 PE				
Pass/Fail (*n* = 1661)				0.82
Pass	196 (11.9%)	412 (25.1%)	1035 (63%)	
Fail	3 (16.7%)	4 (22.2%)	11 (61.1%)	
USMLE Step 1 (*n* = 1296)				
Mean Score	**223.8 (17.3)**	**216 (20)**	**210.2 (18.9)**	**<.001**
Pass/Fail				**<.001**
Pass	180 (15.7%)	308 (26.8%)	660 (57.5%)	
Fail	2 (1.4%)	37 (25%)	109 (73.7%)	
USMLE Step 2 CK (*n* = 895)				
Mean Score	**233 (17.8)**	**232.2 (16.6)**	**226 (18.4)**	**<.001**
Pass/Fail				**0.03**
Pass	127 (15.3%)	232 (27.9%)	474 (56.9%)	
Fail	3 (4.8%)	14 (22.6%)	45 (72.6%)	

^a^All values in Table 4 are presented as N (%) or as Mean (Standard Deviation). Statistically significant associations are shown in bold

We identified a similar effect for passing the USMLE exams on the first attempt (*p* < 0.001 for both Step 1 and Step 2 CK). Specifically, 15.7% of those who passed Step 1 matched into a highly competitive specialty, whereas 1.4% of students that failed it did so. Additionally, 15.3% of those that passed Step 2 matched into a highly competitive specialty as compared to 4.8% of students that failed it. As with the COMLEX exams, we observed a statistically significant difference in mean scores between students that matched into specialties of high- versus low-competitiveness, (13.6 points for Step 1, *p* < 0.001 and 7 points for Step 2, *p* = 0.03; 300-point score scale for both exams). Finally, we observed a statistically significant difference in the mean pre-clinical GPA between students that matched into specialties of high versus low competitiveness (2.4 points on a 100-point scale, *p* < 0.001).

To determine the effect of multiple academic performance factors on matching into highly competitive specialties, we conducted multivariate analysis using a subset of the statistically significant variables in Table 4. Although USMLE Step 1 and 2 CK scores were associated with matching into highly competitive specialties we did not include them in this analysis for the same reasons as described above. The results of the multivariate analysis are shown in Table 5. When considered alone, mean highest MCAT score, pre-clinical GPA, as well as COMLEX Level 1 and Level 2 CE scores were found to have a significant effect. Specifically, for every 5-point increment in mean highest MCAT score, the odds of matching into a highly competitive specialty increased by 19%. Similarly, for every 5-point increment in pre-clinical GPA, the odds of matching into a highly competitive specialty increased by 66%. The magnitude of the effect of Level 1 and Level 2 CE scores was a 4% and a 3% increase respectively in the odds of matching into a highly competitive specialty per 5-point increment in scores. When adjusting for the presence of all these variables, the only factor that still had a significant effect was the COMLEX Level 1 score, with a 4% increase in the odds of matching into a highly competitive specialty for every 5-point increment in score.

**Table 5:** Multivariate analysis of select variables affecting matching into highly competitive specialties for DO 2010–2017

Factor	Unadjusted Odds Ratio	Adjusted Odds Ratio^a^
Pre-admission performance variables		
Mean Highest MCAT Score (increment = 5)	1.19 (1.01, 1.42)	Not significant
Post-admissions performance variables		
Pre-clinical GPA (increment = 5)	1.66 (1.45, 1.89)	Not significant
COMLEX Level 1 Score (increment = 5)	1.04 (1.03, 1.05)	1.04 (1.02, 1.05)
COMLEX Level 2 CE Score (increment = 5)	1.03 (1.02, 1.03)	Not significant

^a^All values in Table 5 are presented as odds ratio (95% confidence interval). Only statistically significant results are shown (*p* < 0.05)

## Discussion

We examined 13 pre- and post-admissions academic performance variables to identify associations with matching into first-choice residency and into highly competitive specialties. Our study has the advantage of a large sample size as well as of examining both institution-specific (undergraduate science and overall GPA, pre-clinical GPA) and standardized (MCAT, COMLEX and USMLE scores) performance factors, with the latter being generalizable and thus potentially applicable to students at other institutions in the United States. Due to differences in selection criteria used for and the process of entering into postgraduate medical education between the United States and other countries such as the United Kingdom and Canada, our findings can only be generalized to other institutions in the United States rather than globally [17, 18].

### Student performance parameters associated with matching into first-choice residency

We found that 53.9% of our graduates matched into their first-choice residency over the eight-year period of the study, which is comparable to the 53% of US allopathic seniors for the 2016 match [10]. When examining factors associated with matching into first choice, we identified an association between pre-clinical GPA and matching into first choice. However, the effect size was small, with < 1-point difference (on 100-point GPA scale) between students that matched into their first choice and those that did not. This may be due to the fact that, unlike standardized licensing exams which allow the comparison of applicants from different institutions, grades can have different meanings at different institutions and, therefore, are not an important selection criterion for residency. We observed a similar effect of pre-clinical GPA using multivariate analysis. Specifically, for each 5-point increment in GPA, the odds of matching into first-choice residency increased by 22%. However, when additional performance factors (mean highest MCAT score, COMLEX Level 2 CE score and passing Level 2 PE) were included in the analysis, this effect was no longer statistically significant. This may be because the large effect of passing COMLEX Level 2 PE on matching into first choice (see below) outweighs the impact of pre-clinical GPA. It is also consistent with residency program directors not citing pre-clinical grades as a criterion for selecting applicants to interview and to rank them post-interview [2].

We also found that passing COMLEX Level 2 PE (a pass/fail exam) on the first attempt is associated with matching into first choice. Furthermore, multivariate analysis demonstrated that passing COMLEX Level 2 PE is the strongest predictor for matching into first-choice residency, increasing the odds of matching by 232% when considered alone and by 268% when accounting for the presence of additional academic performance factors. This is not surprising, since this exam is utilized by program directors to select applicants for interview as well as to rank them post-interview (relative importance 4.2/5 for both) [2]. Additionally, we observed an association of COMLEX Level 2 CE and USMLE Step 2 CK scores, with students that matched into their first choice having higher mean scores (14.1 points for Level 2 CE and 3.9 points for Step 2 CK). A similar effect of COMLEX Level 2 CE score was observed in multivariate analysis, wherein for every 5-point increment in score there was a small effect (1%) of increasing both the unadjusted and adjusted odds of matching into one’s first-choice. Again, this may be expected, since Level 2 CE/Step 2 CK scores are also used by program directors both as interview selection (relative importance 4.1/5) and as post-interview applicant ranking criteria (relative importance 4.1/5) [2]. Surprisingly, we did not identify a similar effect for Level 1 or Step 1 scores. This may be explained by the fact that students select a first-choice residency program (or revise their previous selection) using their Level 1/Step 1 scores as a measure of their competitiveness. Subsequently, they may strive to improve their Level 2 CE/Step 2 CK scores to maximize their chances of a successful match into this program.

Not surprisingly, passing COMLEX Level 2 CE on the first attempt was associated with matching into first-choice residency. Again, this is consistent with “any failed attempts in USMLE/COMLEX” being used as a criterion to select applicants for interview (relative importance 4.6/5) and rank them post-interview (relative importance 4.5/5) [2]. Interestingly, we did not observe a similar effect for passing USMLE Step 2 CK on the first attempt. This may be explained by the fact that passing this exam is not a graduation requirement for our students, rather they opt to take it to increase their competitiveness in the matching process. Furthermore, all AOA-accredited as well as a large number of ACGME-accredited programs use Level 2 CE pass/fail or target scores in applicant selection [2]. Therefore, students that fail Step 2 CK but pass Level 2 CE on their first attempt, can select a first-choice program, and still successfully match solely using their Level 2 CE performance.

Among the pre-admissions academic performance factors tested, we found a statistically significant association of highest MCAT score with matching into first choice. Interestingly, the mean score of students that did not match into their first choice was 0.4 points higher (on a 45-point scale). We observed the same effect in multivariate analysis. Specifically, the odds of matching into one’s first-choice residency decreased for every 5-point increase in mean highest MCAT score, both when considering it alone (19% decrease), and when adjusting for the presence of additional variables (5% decrease). This further confirms the results of our univariate analysis. A previous study has shown that taking the MCAT several times reduces the predictive value of the exam [19]. This, taken together with the fact that the majority of our students have taken the MCAT more than once could account for our observation that the effect is in the opposite direction one would expect.

### Student performance parameters associated with matching into highly competitive specialties

Matching into a highly competitive specialty is a different question. It does not solely involve a student’s subjective assessment of competitiveness or fit with the program, or other criteria such as student geographic preferences, which have been shown to play a role in student ranking of different programs [1, 20, 21]. A large body of work has demonstrated that USMLE Step 1 and/or Step 2 CK scores play an important role in matching into competitive specialties [6–9]. Our findings are consistent with these studies. Specifically, we observed a 62.7-point difference in mean COMLEX Level 1 and a 50.5-point difference in mean Level 2 CE scores of students that matched into specialties of high versus low competitiveness. For USMLE Step 1 and Step 2 CK, we found a 13.6-point and a 6-point difference in mean scores respectively. This difference in mean Level 2 CE and Step 2 CK scores was more pronounced for matching into highly competitive specialties than for matching into first-choice residency. Specifically, it was approximately 3.6-fold larger for Level 2 CE and 1.5-fold larger for Step 2 CK. This difference in magnitude suggests that licensing exam scores are more heavily weighted for matching into highly competitive specialties, than for matching into one’s first choice.

The importance of COMLEX Level 1 and Level 2 CE scores was further confirmed by multivariate analysis. When considered alone, Level 1 and 2 CE scores were associated with an increase in the odds of matching into highly competitive specialties (respectively 4 and 3% per 5-point increment in score). Upon adjusting for the presence of additional variables, COMLEX Level 1 score remained significantly associated, with a 4% increase in the odds of matching per 5-point increment in score. Although this may not appear to be a large effect, given that Level 1 is scored on a 990-point scale, as little as a 30-point increase will result in 25% higher odds of matching into a highly competitive specialty. The fact that COMLEX Level 1 score is the single most important predictor of matching into highly competitive specialties is supported by several lines of evidence. First, it is the only licensing exam score that students are required to submit with their initial residency applications, and since our institutional deadline for taking the exam is September 1st of the fourth year of studies, scores are not available by the September 15th deadline for submission of initial residency applications. Additionally, since, based on our experience, students typically submit their Level 2 CE score by November or in December (in the middle or towards the end of interview season), Level 1 is frequently the only score available to program directors when they select applicants to interview and during the interview process. Accordingly, COMLEX Level 1 is almost universally used by program directors to select candidates to interview (93% cite it as an important factor) and remains one of the most commonly used criteria to rank candidates post-interview (78% of program directors cite it as important) [2]. If candidates’ Level 2 CE scores are available during the interview selection process they are also used, albeit not as frequently as Level 1 scores (83% of program directors cite Level 2 CE scores as an important factor) [2]. Finally, since COMLEX Level 2 CE scores are required by programs to rank candidates they have already interviewed (most programs have a December deadline for submission of Level 2 CE scores which is towards the end of interview season), they play a secondary role in the matching process, with Level 1 scores being the most important determinant. Taken together, these observations also suggest an explanation as to why we did not observe a statistically significant association of Level 2 CE scores with the adjusted odds of matching into highly competitive specialties.

Furthermore, passing licensing exams on the first attempt was a critical factor for matching into highly competitive specialties. We found that 0% of students that failed COMLEX Level 1 and only 4.4% of those that failed Level 2 CE matched into highly competitive specialties. In comparison, 12.6% of students who passed Level 1 and 12.4% of those who passed Level 2 CE on the first attempt matched into specialties of high competitiveness. Similarly, for USMLE, only 1.4% of students that failed Step 1 and 4.8% of those that failed Step 2 on the first attempt matched into highly competitive specialties, whereas 15.7 and 15.3% of those that passed Step 1 and Step 2 CK on the first attempt respectively, did so. These findings are consistent with the 2016 NRMP Program Director survey where (on average) 96% of program directors in highly competitive specialties indicated that they would never/seldom consider applicants that failed USMLE Step 1 or Step 2 CK on the first attempt. In addition, 80.5 and 88.5% (on average) of program directors in low-competitiveness specialties stated they would never/seldom consider applicants that failed Step 1 or Step 2 CK respectively [2].

In contrast to our findings regarding matching into first-choice residency, we did not identify a statistically significant association of passing COMLEX Level 2 PE (a pass/fail exam) on the first attempt with matching into highly competitive specialties. A likely reason for this observation is that Level 2 PE is considered as a minimum competency exam by program directors of highly competitive specialties.

In addition to licensing exams, we identified a statistically significant association of pre-clinical GPA with matching into highly competitive specialties. However, the effect size was small with a 2.4-point difference between the mean GPA of students that matched into specialties of high versus low competitiveness. The fact that the effect size of pre-clinical GPA was small compared to that of licensing exam scores suggests that, since grades are not standardized among different medical schools, they are not as useful in comparing applicants to residency programs. In contrast, scores on standardized licensing exams allow program directors to compare diverse applicants from across the country. The results of multivariate analysis further support this, since, although pre-clinical GPA affected the unadjusted odds of matching into highly competitive specialties (66% increase per 5-point increment in GPA), there was no statistically significant association with the adjusted odds, indicating that pre-clinical GPA is not a predictor of matching, particularly when considered in context with COMLEX Level 1 score.

Finally, using univariate analysis, we did not observe an association between any pre-admissions academic performance variables, such as highest MCAT score or science GPA, with matching into highly competitive specialties. This is not surprising, since, although undergraduate GPA and MCAT scores allow for successful admission to medical school, they are not part of residency applications and thus are not available to program directors. When highest mean MCAT score was included in the multivariate analysis, it was associated with a 19% increase in the unadjusted odds of matching into highly competitive specialties per 5-point increment in score. However, when adjusting for the presence of additional performance factors (COMLEX Level 1, Level 2 CE and pre-clinical GPA), this effect was no longer statistically significant. A large body of work has demonstrated a small to medium predictive validity of MCAT scores for licensing exam performance, particularly for USMLE Step 1 [22, 23], and since USMLE Step 1 is a key determinant for matching into highly competitive specialties (as is COMLEX Level 1 according to the present study) [2, 7, 9, 24], this may account for the observed association of MCAT score with the increased unadjusted odds of matching into highly competitive specialties. However, since the effect of MCAT score on matching is indirect via its effect on Step 1/Level 1 scores, it is no longer statistically significant when considered in the context of COMLEX Level 1, which we found to be the single strongest predictor of matching into highly competitive specialties.

### Limitations of the study

This study has a number of limitations. First, the relative competitiveness of specialties may slightly change from year to year, in part due to the variability in the number of applicants and positions available [25]. Our study population consisted of students that matched into residency from 2010 to 2017, however, we used the 2017 Careers In Medicine® competitiveness ratings.. To estimate the effect of this limitation, publicly available match data from the National Resident Matching Program (NRMP) annual residency match reports 2010–2017, as well as Electronic Resident Application Service (ERAS) data, that provide detailed information for each specialty on the number of available positions, number of applicants, number of positions filled and number of positions filled by US seniors, was used to determine the specialty fill rate by US seniors, which is a measure of its competitiveness [26]. This analysis revealed small year-to-year fluctuations in fill rate for the specialties included in this study during the eight-year study period. However, these fluctuations were not sufficient to result in any changes in competitiveness levels for any of these specialties during the study period.

A second limitation is that our students can apply for, and thus, match into either AOA- or ACGME-accredited residency programs, which may differ in competitiveness within the same specialty. However, this difference is not sufficient to cause a given specialty to move from one competitiveness category to another depending on whether the program is AOA- or ACGME-accredited. Since licensing exam scores are a key determinant in matching [2], if ACGME-accredited programs are more competitive than AOA programs within the same specialty, one would expect licensing exam scores of students matching into the former to be higher than those matching into the latter. This will result in an increase in mean exam scores than if it were the other way around. Since approximately the same percentage of students matched into ACGME versus AOA-accredited programs in the high and medium competitiveness categories (high: 53.8% ACGME, 44.2% AOA; medium: 51.9% ACGME, 42.5% AOA), mean licensing exam scores in each group will increase by the same extent, and the magnitude of the statistically significant differences we observed between these two groups is not affected by whether students matched into ACGME or AOA programs. In the low competitiveness category, a higher percentage of students matched into ACGME-accredited programs (62% ACGME, 31.9% AOA). Therefore, mean licensing exam scores in this group will be higher and this will attenuate the magnitude of the difference we observed between the high and low competitiveness, as well as between the medium and low competitiveness groups.

Third, whether students matched into their first-choice residency program is self-reported and has not been independently validated. However, students have no incentive or reward to falsify this data. The Electronic Residency Application System (ERAS) which holds the information on first, second or third choice match, does not officially release this information, thus all medical schools in the United States must rely on self-reported data [27]. Additionally, in the present study, data on the specific specialty and residency program that students matched into was also self-reported. However, this was also officially validated by the Office of the Registrar at our institution, and we did not identify any inconsistencies between the self-reported and Registrar data. Thus, there is also no reason to doubt the validity of the self-reported data on first-choice match.

An additional limitation consists of the fact that students may revise their original first-choice residency selection using their COMLEX Level 1 or USMLE Step 1 score as a measure of their competitiveness, in order to maximize their chances of a successful match. This would result in an increased number of students matching into their first-choice program. However, this would not be unique to our institution, rather it would likely occur in osteopathic and allopathic institutions throughout the U.S., particularly in light of the fact that students are constantly advised of the importance of Level 1/Step 1 scores for matching into residency.

The fact that there is significant heterogeneity in the pre-clinical grading systems currently employed in U.S. medical schools (pass/fail, honors/pass/fail, numeric grades among others) limits the applicability of our findings with regards to the effect of pre-clinical GPA on matching into first-choice residency and competitive specialties. However, since there is no national standard for grading systems, this limitation applies to any study examining pre-clinical GPA and is not exclusive to our study.

Since this study focuses exclusively on students from one institution, the effect of medical school quality or competitiveness on matching into residency could not be assessed. A number of studies have shown that medical school quality (as measured by the widely accepted “U.S. News and World Report” rankings which, among other factors, capture the acceptance rate or competitiveness of a medical school) plays a role in matching into certain highly competitive specialties such as ophthalmology, otolaryngology and orthopedic surgery [7–9, 28]. Additionally, “graduate of a highly-regarded U.S. medical school” is used as a criterion by program directors for selecting applicants to interview as well as for ranking applicants that have already interviewed [2]. Furthermore, less competitive medical schools tend to attract and admit applicants with lower MCAT scores. Since MCAT scores have been shown to possess a small to moderate predictive validity for licensing exam scores, particularly for USMLE Step 1, students in less competitive medical schools are more likely to score lower on their licensing exams, which will in turn decrease their likelihood of matching into highly competitive specialties [22, 23]. The most current “U.S. News and World Report” rankings place our school in the bottom 25% of the 120 ranked allopathic and osteopathic medical schools, therefore, given all of the above, one would expect fewer of our students to match into highly competitive specialties. In fact, during the eight-year study period, only 12% of our graduates did so. However, the fact that our school is in the bottom 25% of ranked schools does not affect the validity of our findings. A study from the University of Minnesota Medical School, which is a top 50 ranked institution, identified an association of licensing exam scores and residency specialty match, with students matching into specialties such as dermatology (highly competitive) having the highest mean scores and students matching into family medicine (low competitiveness) having the lowest mean scores, which is consistent with our findings [24].

Finally, we considered a large and comprehensive number of academic variables (which represent “hard skills”), however we did not consider any non-academic factors (“soft skills”), such as interpersonal skills and performance on the interview, which are much more difficult to quantitate but which play an important role in programs ranking applicants and can thus affect matching [2].

## Conclusions

This study demonstrated the importance of passing licensing exams on the first attempt, as well as of exam scores, for matching both into first-choice residency and into highly competitive specialties. However, the difference in scores was more pronounced for matching into highly competitive specialties than it was for matching into first-choice residency. Our findings are in agreement with existing literature and supported by the fact that these exams are universally used by program directors for selecting applicants for interview and for post-interview ranking. Our results can help faculty better advise students, inform curricular design to optimize matching, as well as understand student risk factors for not matching.

## Data Availability

The dataset generated and/or analysed during the current study is not publicly available due to the ratio of disparate data points associated with a relatively small study cohort, which, with the right data techniques and in the wrong hands, could potentially lead to the identification of subjects and to the breach of participant confidentiality, but the dataset is available from the corresponding author on reasonable request.

## Abbreviations

AACOM: American Association of Colleges of Osteopathic Medicine
AAMC: American Association of Medical Colleges
ACGME: Accreditation Council for Graduate Medical Education
AOA: American Osteopathic Association
CE: Cognitive Evaluation
CK: Clinical Knowledge
COM: College of Osteopathic Medicine
COMLEX: Comprehensive Osteopathic Medical Licensing Examination
CS: Clinical Skills
DO: Doctor of Osteopathic Medicine
ERAS: Electronic Residency Application System
GME: Graduate Medical Education
GPA: Grade Point Average
MCAT: Medical College Admission Test
MD: Medical Doctor
NRMP: National Resident Matching Program
PE: Performance Evaluation
SAS: Statistical Analysis System
SOM: School Of Medicine
USMLE: United States Medical Licensing Examination
